# Linear indium atom chains at graphene edges

**DOI:** 10.1038/s41699-023-00364-6

**Published:** 2023-01-25

**Authors:** Kenan Elibol, Toma Susi, Clemens Mangler, Dominik Eder, Jannik C. Meyer, Jani Kotakoski, Richard G. Hobbs, Peter A. van Aken, Bernhard C. Bayer

**Affiliations:** 1 University of Vienna, Faculty of Physics, Boltzmanngasse 5, A-1090 Vienna, Austria; 2 Max Planck Institute for Solid State Research, Heisenbergstrasse 1, 70569 Stuttgart, Germany; 3 Institute of Materials Chemistry, Technische Universität Wien (TU Wien), Getreidemarkt 9/165, A-1060 Vienna, Austria; 4 Institute for Applied Physics, University of Tübingen, Auf der Morgenstelle 10, 72076 Tübingen, Germany; 5 Centre for Research on Adaptive Nanostructures and Nanodevices (CRANN) and the SFI Advanced Materials and Bio-Engineering Research Centre (AMBER), Dublin 2, Ireland; 6 School of Chemistry, Trinity College Dublin, The University of Dublin, Dublin 2, Ireland

**Keywords:** Two-dimensional materials, Electronic properties and devices

## Abstract

The presence of metal atoms at the edges of graphene nanoribbons (GNRs) opens new possibilities toward tailoring their physical properties. We present here formation and high-resolution characterization of indium (In) chains on the edges of graphene-supported GNRs. The GNRs are formed when adsorbed hydrocarbon contamination crystallizes via laser heating into small ribbon-like patches of a second graphitic layer on a continuous graphene monolayer and onto which In is subsequently physical vapor deposited. Using aberration-corrected scanning transmission electron microscopy (STEM), we find that this leads to the preferential decoration of the edges of the overlying GNRs with multiple In atoms along their graphitic edges. Electron-beam irradiation during STEM induces migration of In atoms along the edges of the GNRs and triggers the formation of longer In atom chains during imaging. Density functional theory (DFT) calculations of GNRs similar to our experimentally observed structures indicate that both bare zigzag (ZZ) GNRs as well as In-terminated ZZ-GNRs have metallic character, whereas in contrast, In termination induces metallicity for otherwise semiconducting armchair (AC) GNRs. Our findings provide insights into the creation and properties of long linear metal atom chains at graphitic edges.

## Introduction

Graphene is of significant importance for future applications including next-generation integrated circuits^1,2^. The absence of a bandgap in graphene limits, however, its use in nanoelectronic and optoelectronic devices^3–6^. The fabrication of one-dimensional confined graphene structures—graphene nanoribbons (GNRs)—can enable tunable bandgaps^1,7–9^. The electronic nature of GNRs depends on their width, chirality and edge structure^1,10,11^. When the edges are oriented along zigzag (ZZ) or chiral directions, the ribbons are metallic but a magnetic ordering emerges due to localized edge states^12^. An external transverse electric field may alter their electronic properties, inducing half-metallicity^13^. Instead, narrow GNRs with armchair (AC) edges have a bandgap, which depends strongly on the ribbon width^14,15^.

The functionality of GNRs can be enhanced via doping and edge modifications^16,17^. Theoretical studies have shown that the electronic and magnetic properties of GNRs are modulated with the adsorption of metal atoms such as Cu, Fe, Co, Ni, Ag, Au, Mn and Pt at their edges^18–21^. Boron (B) doping at the edges of ZZ-GNRs results in half-metallic behavior, while nitrogen (N) atoms induce metallic behavior in ZZ-GNRs^22,23^. The antiferromagnetic ZZ-GNRs become ferromagnetic with the adsorption of C, B and N atoms at their edges^24^. Additionally, Ni atoms lying at the edges of ZZ-GNRs change their magnetization^25^. Compared to these ample theoretical studies, little experimental work has however explored the edge decoration of GNRs. So far, several elements in the form of *individual* metal atoms (e.g. Fe, Cr, Cu, Sn, Ni, Al, Pt and Au) attached to graphene edges have been revealed via transmission electron microscopy (TEM)^26–35^. Via single-walled carbon nanotube (SWNTs) opening, also the creation and atomic-resolution characterization of sulfur (S)-terminated GNRs by TEM has been reported^3,16^. In contrast, experimental synthesis and high-resolution characterization of *metal atom chains* at GNR or graphene edges remains elusive.

To address this gap, we report here the experimental creation of linear indium (In)-atom chains along the edges of graphene-supported GNRs. The element In is interesting in this regard since as an atomic adsorbate, it modifies the electronic properties of graphene^36^ and also is a potent single-atom catalyst for, e.g., CO_2_ reduction when anchored as single atoms onto carbon materials^37,38^. We form these GNRs in ultra-high-vacuum (UHV) conditions in a scanning transmission electron microscope (STEM) via laser-induced high-temperature crystallization of adsorbed hydrocarbons to form small GNR-like patches of a second layer of graphene supported on suspended monolayer chemical vapor deposited (CVD) graphene membranes, following prior work in the literature^39,40^. The In-atom chains are then formed without breaking the vacuum via in-situ physical vapor deposition (PVD) of In onto the GNR/CVD graphene samples and subsequent laser annealing.

The existence of In-terminated near-ZZ and near-AC edges of GNRs is verified by atomic-resolution STEM imaging and electron energy-loss spectroscopy (EELS). Detailed structures of In-terminated near-ZZ- and near-AC-oriented GNR edges are further confirmed through STEM image and density functional theory (DFT) simulations. The electron-beam-induced dynamics of In atoms along the near-ZZ and near-AC edges of the GNRs are monitored via sequential atomic-resolution STEM imaging. Finally, DFT simulations indicate that such In-atom termination can modify the electronic properties of ZZ- and AC-GNRs. While there is limited difference in the electronic structures of the bare and In-terminated ZZ-GNRs, In decoration strongly alters the lowest unoccupied bands as well as dopes the semiconducting AC-GNR. Our data thus provides insights into the creation and properties of long linear metal atom chains at graphene and GNR edges.

## Results

### Formation of In-atom chains

We have recently established the in-situ synthesis and atomic-resolution characterization of single In atoms and few-atom In nanoclusters anchored on monolayer graphene^41^. Here, we apply the same methodology for the creation of In-atom chains at the edges of GNRs (see Methods). We note that for the majority of the sampled areas, we observe anchored In atoms and few-atom In nanoclusters as described in our prior work^41^, while for few regions on the same samples, we observe the here reported In-decorated GNRs. As earlier described^41^, the experiments presented in this work are performed in a coupled STEM setup, which involves the microscope and an in-situ UHV preparation system comprising deposition and laser-annealing chambers, enabling sample transfer between preparation steps and imaging without ambient air exposure^41,42^. In short, the sample preparation is as follows: First, a suspended CVD monolayer graphene membrane is loaded into UHV, followed by laser annealing of the membrane to remove adsorbed hydrocarbons that are typically present from ambient air exposure^43,44^. We find that a small fraction of these adsorbed hydrocarbons does not desorb, but instead crystallizes into small ribbon-like patches of an additional layer of graphene under laser irradiation, similar to prior reports in the literature^39,40^. Subsequently, we use PVD to deposit In onto the membrane and then subject the sample to a second laser anneal, which leads to diffusion of the deposited In across the sample surface^41^. Finally, the samples are imaged using STEM.

**Fig. 1:**
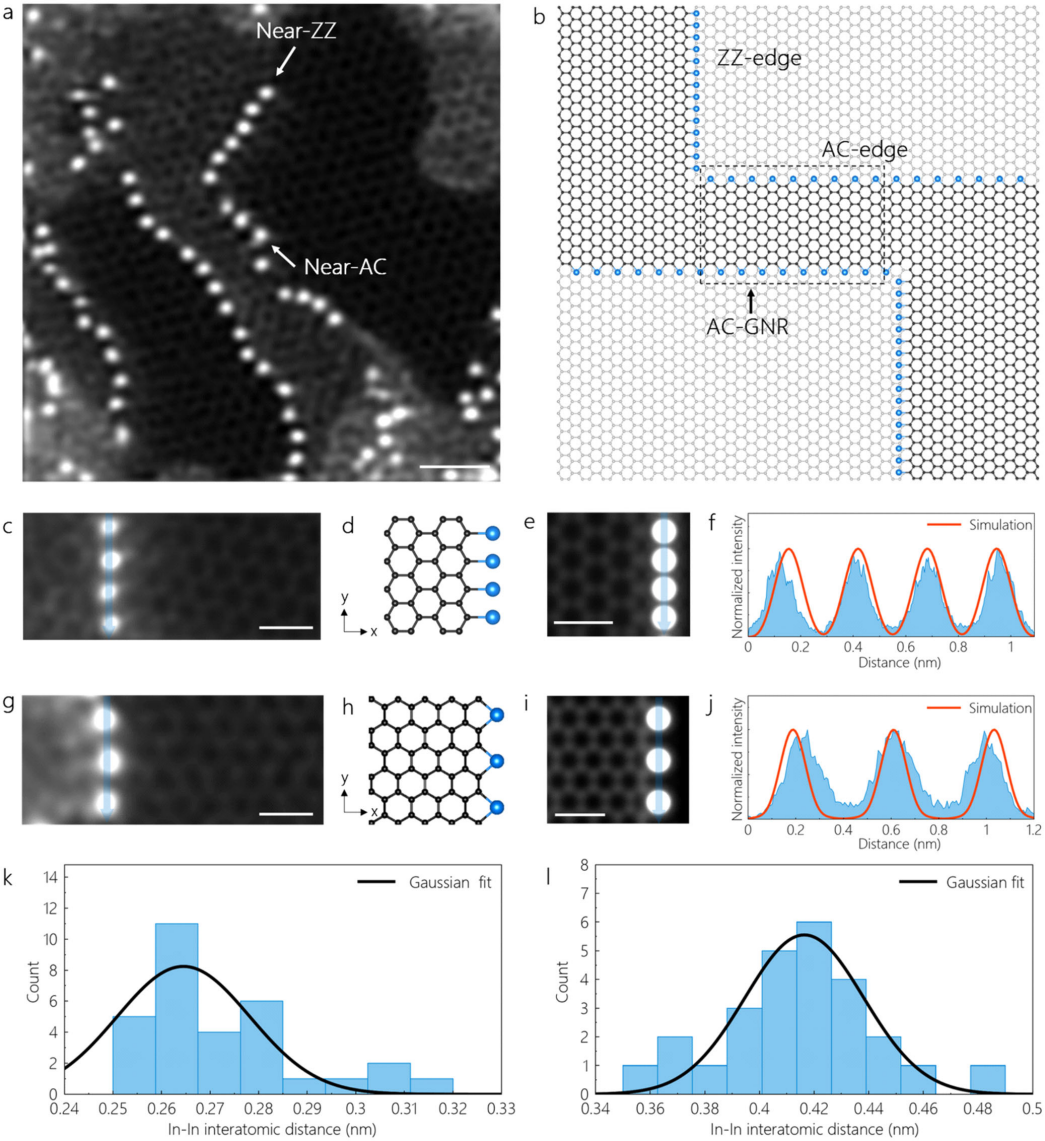
In-terminated near-ZZ and near-AC graphene edges. **a** MAADF-STEM image of In-terminated graphene edges of a GNR formed on the supporting graphene monolayer. The image is double Gaussian filtered (raw image is shown in Supplementary Fig. 1b). The scale bar is 1 nm. **b** A simplified schematic showing In-terminated graphitic edges of GNRs on graphene, corresponding to an idealized model of the structure in (**a**). **c**, **g** MAADF images of the near-ZZ and near-AC graphene edges terminated with In atoms. **d**, **h** DFT-relaxed models of In-terminated ZZ- and AC-edges (C and In atoms are represented by black and blue colored spheres, respectively) and (**e**, **i**) their corresponding simulated MAADF images. The images are double Gaussian filtered (the raw images are shown in Supplementary Fig. 4). **f**, **j** Intensity profiles measured along the semi-transparent blue lines on the In-terminated (near-)ZZ and (near-)AC-edges shown in the experimental and simulated MAADF images. The scale bars in (**c**, **e**, **g**, **i**) are 0.5 nm. **k**, **l** Histograms showing the experimentally measured interatomic In-In distance along near-ZZ and near-AC GNRs, respectively.

Figure 1a displays a medium-angle annular dark-field (MAADF) STEM image of the structures arising from our preparation. In Fig. 1a, we find a small laser-crystallized graphene patch on the continuous monolayer graphene membrane. This ribbon-like patch notably has what appear to be In-terminated edges, as indicated by the bright MAADF signal at the ribbon edges and as further confirmed below. The only few-nm width of this graphene patch motivates us to call it a GNR. However, due to the imperfect preparation process, the termination of the edges of the GNR in Fig. 1a is not straightforward to assign. Atomic-resolution STEM images reveal that the crystallinity of the GNR on graphene is imperfect and it contains structural defects (Supplementary Fig. 1a). Fourier-transform (FT) data recorded on the laser-induced GNR and the adjacent supporting monolayer graphene areas shows the co-existence of two slightly rotated graphitic reflections (Supplementary Fig. 1c–e). Together with the real space MAADF data in Supplementary Fig. 1a this suggests that the GNR is almost AA-stacked with respect to the supporting monolayer graphene membrane with a small misorientation of ~2°. The structural disorder in the GNRs and their misorientation with respect to the continuous graphene support hinders exact edge assignments. Combined, the atomic-resolution images and the FT data do, however, indicate that the GNR edges labeled in Fig. 1a are close to ZZ and close to AC directions, respectively. Thus, for the purposes of further discussion and analysis, we term these as “near-ZZ” and “near-AC”, respectively.

In Fig. 1a, we find the In atoms to be lined up along these near-ZZ and near-AC graphene edges of the GNR. Figure 1b correspondingly shows a schematic exhibiting In-decorated graphene ZZ- and AC-edges of an idealized model GNR located on a graphene monolayer. The identity of the In atoms at the GNR edges is confirmed by the EELS spectra shown in Supplementary Fig. 2. Compared to prior experimental literature on metal-decoration of graphene edges, which had only reported isolated metal atoms^26–30^, the decoration of graphitic edges observed here is by many metal atoms lined up along edges. However, a small amount of In also remains in the amorphous residual (hydro-)carbon adsorbate areas on the bare graphene (Fig. 1a, left).

Figure 1c shows a close-up MAADF image of the near-ZZ graphene edge decorated with In atoms. To corroborate the edge termination, we simulated a ZZ graphene edge terminated with In in Klein configuration^45,46^ using DFT (Fig. 1d), and then performed a MAADF image simulation of the DFT-relaxed model (Fig. 1e). When comparing the In-atom spacing as well as the MAADF intensity derived from the experimental and simulated MAADF images (Fig. 1f), we find a good agreement in the measured and simulated intensity profiles. To determine the spacing, we measured a histogram of experimental In-In distances for the near-ZZ edge (Fig. 1k). By fitting a Gaussian lineshape, we estimate the In-In distance along the experimental near-ZZ GNRs to be 0.27 ± 0.02 nm, which matches our DFT-relaxed model remarkably well (0.26 nm). This corroborates our assignment of In atoms in Klein configuration terminating this near-ZZ graphene edge of the GNR.

We performed a similar analysis for the near-AC edges (see Fig. 1g–j, l), which suggests for near-AC In decoration in a bivalent configuration^46^. The slight apparent mismatch in the spacing of the experimental and simulated MAADF line profiles (see Fig. 1f, j) may be ascribed to poor crystallinity of GNRs and the misorientation of the actual near-AC-edge in this case. A statistically more robust histogram of In-In distances on the AC-GNR (Fig. 1l) yields 0.42 ± 0.03 nm, which is in good agreement with our DFT-relaxed model (0.42 nm). Overall, these findings support In atom termination on the near-AC edge of the GNR with the In in a bivalent configuration.

Prior literature indicates that also curvature induced by folding can alter the local chemical reactivity of graphene membranes^47^. Interestingly, we observe here that In atoms do not form an atomic chain at the highly symmetric edge of a graphene layer folded upon itself to form a local bilayer (see Supplementary Fig. 3).

### Dynamics of In atoms at GNR edges

**Fig. 2:**
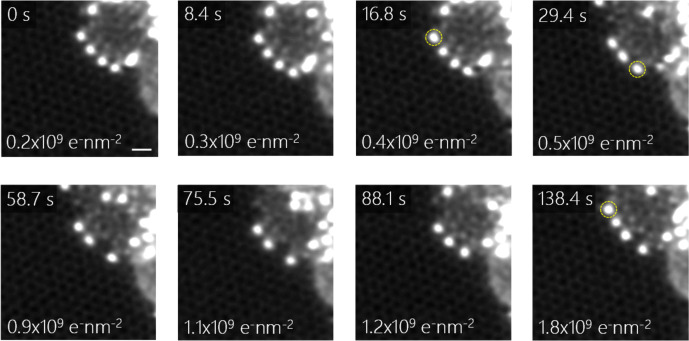
Dynamics of In atoms at AC-edges. MAADF-STEM image series of an In-terminated graphene AC-edge, with cumulative irradiation doses and corresponding irradiation times indicated. The images are double Gaussian filtered, and the scale bar is 0.5 nm.

To study the impact of energetic electron irradiation in STEM on the spacing of In atoms, we show images acquired during electron-beam irradiation in Fig. 2. Here, the images are sequentially acquired, thus corresponding to increasing irradiation dose. No obvious changes occur until a dose of 0.4 × 10^9^ e^–^nm^−2^. With an increase of dose up to 0.5 × 10^9^ e^–^nm^−2^, the In atom marked by the dashed yellow circle in the third frame is ejected from the near-AC GNR edge. Subsequently, another In atom marked by a dashed yellow circle shown in the fourth frame moves to the neighboring site when the electron dose reaches 0.9 × 10^9^ e^–^nm^−2^. The same In atom then moves back to its previous location at a dose of 1.2 × 10^9^ e^–^nm^−2^. A further increase of the dose also enables capturing additional In atoms at the edge of the GNR (see In atom marked by the dashed yellow circle in the last frame). Consequently, electron-beam irradiation induces variations in the interatomic distances of the In-atom chains due to the migration of atoms along the near-AC edge. Our prior work indicated significant diffusion of In across graphene membranes faster than our imaging timescales even at room temperature due to the combination of In’s low melting point (~160 °C) and low vapor pressure^41,48^. We thus also here rationalize the capture of additional In atoms by In diffusion and then preferential decoration of chemically highly reactive sites such as graphitic edges by these atoms. The continuous graphene support membrane stabilizes the GNR edges and acts as a platform for mobile In atoms moving on its surface.

**Fig. 3:**
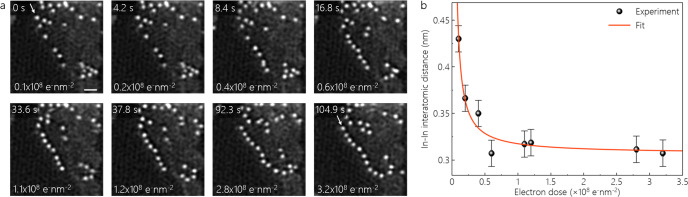
Dynamics of In atoms at ZZ-edges. **a** MAADF-STEM image series of an In-terminated graphitic ZZ-edge acquired at increasing cumulative irradiation doses and corresponding times indicated. The images are double Gaussian filtered and the scale bar is 1 nm. The near-ZZ-edge is indicated by the white arrows shown in the first and last frames. **b** The interatomic In-In distances along the near-ZZ GNR as a function of electron dose. The error bars show the standard error of the mean.

Figure 3a shows electron-beam-induced dynamics and formation of long In-atom chains along a near-ZZ GNR edge. Since these images were acquired at a high scan rate to capture the dynamics, the image resolution is rather poor. To verify the edge termination, we measured the spacing of the In atoms at the chain formed along the edge, again comparing it to DFT-relaxed models (see Supplementary Fig. 5). At low electron doses, there are only a few In atoms lined up at the near-ZZ edge. Notably, at electron doses reaching 0.4 × 10^8^ e^–^nm^−2^, the edge gains more In atoms and the chain grows further. As discussed above, this again suggests that diffusing In atoms from outside of the field-of-view can be captured at the edges during imaging. More In atoms are attached to the GNR edge at higher electron doses. The explicit increase in the number of In atoms is obvious at an electron dose of 0.6 × 10^8^ e^–^nm^−2^. The higher the cumulative electron dose, quantitatively, the more atoms does the near-ZZ-edge gain and the longer the chain becomes, culminating in the last frame corresponding to an electron dose of 3.2 × 10^8^ e^–^nm^−2^. Figure 3b plots the corresponding interatomic In-In distances along this near-ZZ edge as a function of electron dose. The initial decrease in In-In distance reflects the increasing decoration of the GNR edge by In. Notably, the In-In distance remains also after extended electron dose and a fully decorated edge larger (~0.31 nm) compared to Fig. 1 (~0.26 nm). We ascribe this to the imperfection and misorientation of the particular near-ZZ in Fig. 3.

The finding that In-In distances for near-ZZ can vary to some extent (compare Fig. 1 versus Fig. 3) suggests that In termination of GNR is not limited to perfect AC and ZZ edges (models in Fig. 1) but can also occur at chiral GNRs (Figs. 2 and 3) with certain misorientations from AC and ZZ, respectively. Unfortunately, the HAADF data of the GNRs in Figs. 2, 3 is not of sufficient quality to fully assess for us the exact chiral indices of their near-AC and near-ZZ edges.

The maximum lengths of linear In chains along near-ZZ, near-AC edges of GNRs are measured to be ~3.1 nm and ~2 nm, respectively. For (kinked) edges comprised of both near-ZZ and near-AC sections (“mixed edges”) we measure the longest In chain with length of ~4.9 nm.

### The electronic properties of In-decorated GNRs

**Fig. 4:**
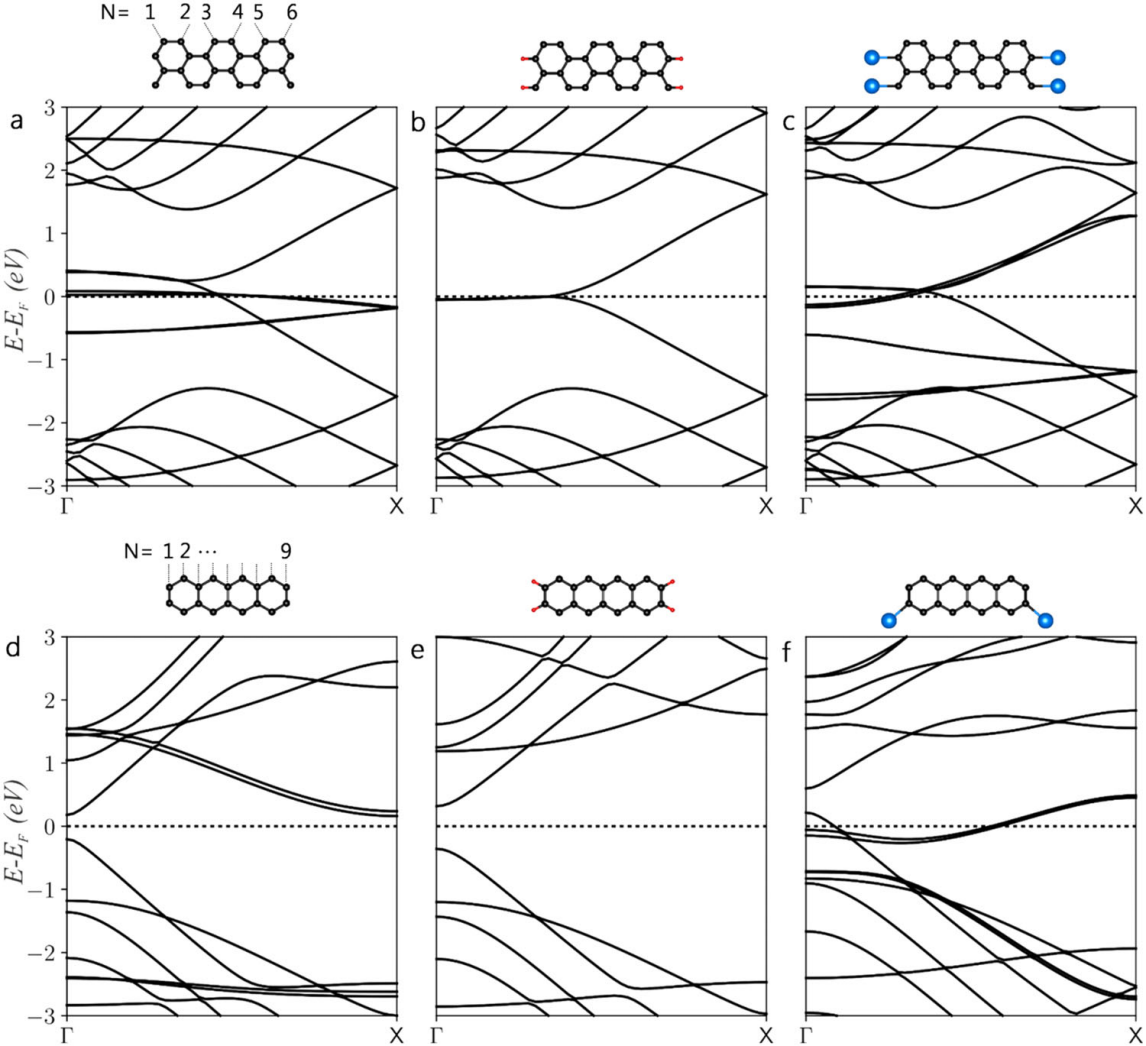
The electronic band-structures. The electronic band-structures of bare, H-terminated and In-terminated (**a**–**c**) ZZ (*N* = 6) and (**d**–**f**) AC (*N* = 9) GNRs calculated by DFT along the Γ-X direction which corresponds to conduction direction in the periodic real-space direction along the ribbons. The unit cells of the DFT-relaxed models used in the band-structure simulations are shown above, where the C, H and In atoms are represented by black, red and blue colored spheres, respectively.

Having experimentally established that graphene edges in supported GNRs can be decorated by In atom chains along their edges, we now turn to investigate how the electronic properties of GNRs could be affected by such decoration. In particular, in Fig. 4 we show the DFT-calculated band structures of bare, commonly suggested H-terminated and here reported In-terminated ZZ- and AC-GNRs of widths *N* = 6 and *N* = 9, respectively. Here, *N* is the total number of zigzag chains across the ZZ-GNR whereas it is the total number of dimer lines across the AC-GNR. The reason we chose the supercell sizes of *N* = 6 and *N* = 9 is that their respective widths are close to our experimentally observed GNR widths of ~1 nm (Fig. 1a). Figure 4a shows the characteristic flat bands of bare ZZ-GNRs alongside two edge states that are suppressed by H saturation as seen by comparison to Fig. 4b. Earlier reports have shown that the nonbonding orbitals localized at the edges of ZZ-GNRs constitute these flat bands and give rise to a steep peak in the density of states (DOS)^49^, which depend on the ribbon width^50^. When the ZZ-GNR is terminated by In atoms, a rearrangement of these edge states corresponding to the highest occupied bands can be observed especially towards X, but the structure remains metallic (see Fig. 4c). Unlike ZZ-GNR, the bare AC-GNR is a semiconductor with a small bandgap (see Fig. 4d), which is reduced with increasing ribbon width^51,52^. Interestingly, when its edges are terminated with In atoms (see Fig. 4f), the lowest unoccupied band that is suppressed by H saturation (Fig. 4e) is significantly modified especially towards Γ, and the semiconducting AC-GNR is doped into a metallic state. No prior calculations for In decoration are available, but iron (Fe) and titanium (Ti) adsorption at the AC-edges of GNRs has previously been predicted to turn them into half-metals^53^.

Besides band structures, we also study the energetics of In-chain formation on GNR edges by DFT (Supplementary Fig. 6 and Supplementary Tables 1, 2) using a supercell obtained by repeating the unit cell by six times along the ribbon length, inserting additional In atoms, and relaxing the structures to calculated their total energies. By comparing these to the bare ribbon, we can estimate the energy gain for each additional In atom saturating the highly reactive edges.

For the AC-GNR (Supplementary Fig. 6 and Supplementary Table 2), each In atom added to the edge results in a relatively larger energy gain—until the six most favorable adsorption sites are saturated, after which the energy gain starts to decrease. This suggests that full decoration of the AC edge by In is energetically favored, but if additional edge sites are available, this relatively sparse decoration is the overall energy minimum. Throughout the simulated formation sequence until six added atoms, all In atoms show a bivalent bonding to the AC edge (except for four In atoms where their bonding dimerizes), same as in the final bivalent In configuration that we also experimentally observe (Figs. 1, 2). More In atoms can be accommodated, but at diminishing energetic returns.

For ZZ-GNRs (Supplementary Fig. 6 and Supplementary Table 1), we find that the addition of each In atom to the graphene edge also leads to an energy gain up to the fully saturated edge, suggesting that formation of In chains at ZZ edges is energetically favored. Interestingly however, the energy gain for each atom added is in this case reducing in relative terms with increasing In number. While the final configuration of the fully decorated edge from DFT in Supplementary Fig. 6 is similar to the experimentally observed Klein configuration in Figs. 1, 3, we note that for lower In numbers the non-saturated ZZ-edge shows different intermediate bonding types than the fully saturated edge. Individual In atoms, when there is space available at the non-saturated ZZ-GNR edge, appear to prefer to bond to multiple edge C atoms in a mixed (partly bivalent) bonding type. We have not experimentally observed such intermediate states in our experiments, but note that this may be related to the limited number of intermediate states that we have captures in our in-situ formation data that has a limited time resolution.

Since the GNRs in our experimental data are rather defective, we have also tentatively calculated the impact of exemplary defects within the GNR on the In adsorption at edges adjacent to the defects. As shown in Supplementary Fig. 7, the energy gain from adding an In atom to the edge of AC-GNR with a defect is in some cases slightly greater than that of a single In atom attached to a perfect AC-GNR (Supplementary Fig. 6). This suggests that defects in the GNRs may even be conducive towards In decoration of the GNR edges, although certainly the configuration as a whole is not energetically favorable due to the defect.

Overall, these energetic considerations support the finding that In decoration of both AC and ZZ GNR edges is favored and DFT predicts the same final In configurations for the fully In-decorated GNR edges that we have experimentally observed.

We finally turn to discuss the limitations of this study: While we have demonstrated the viability of In-chain decoration of GNRs under UHV conditions with stability at room temperature, the presented structures require additional steps towards future application screening. These steps in particular include i. improving the scalability and the selectivity of the preparation route towards In-chain/GNR structures (i.e. beyond their sparse formation as in this proof-of-existence study) and ii. assessing the stability of the structures under oxidative or reductive conditions in future operando-type work, as typically encountered in, e.g., realistic GNR device fabrication process flows or possible catalysis applications.

## Discussion

In summary, we demonstrate long linear In-atom chains along edges of GNR-like graphitic structures supported on continuous graphene monolayer membranes. The In-terminated near-ZZ and near-AC graphitic edges are imaged at a high spatial resolution using aberration-corrected STEM. All the structures observed experimentally are verified via DFT and image simulations, and the identity of In is confirmed by EELS. The electron-beam irradiation leads to the displacement of In atoms along the edges promoting the formation of long attached atomic In chains. The electronic properties of similar In-terminated GNRs are predicted by DFT simulations, whereby In atoms adsorbed at the edges of ZZ-GNRs do not induce a distinct change in the electronic structure, but doping and an explicit reduction in the bandgap of semiconducting AC-GNR is observed, when their edge is terminated by In atoms. Our findings experimentally extend the concept of metal decoration of graphene edges from isolated atoms to atomic chains along the edges. Thus, not only single atoms can be prepared on graphene edges (as in prior literature^26–30^), but also metal-atom chains can decorate them. This may be of future interest in tuning electronic properties of GNRs by metal-atom chain decoration. Our data also suggests that In particularly well lends itself for such atom chain decoration of graphene edges. Because atomic In is a potent single-atom catalyst for, e.g., CO_2_ reduction^37^, we suggest that it could be also attractive in future work to explore how such metal atom chains fare in catalysis^54^.

## Methods

### Samples

We used commercial graphene samples grown via chemical vapor deposition and transferred onto Quantifoil TEM grids (Graphenea Inc.).

### STEM and EELS measurements

STEM imaging was performed using a Nion UltraSTEM100 operated at a 60 kV accelerating voltage in UHV (~10^−9^ mbar). The collection angles of the high-angle annular dark-field (HAADF) and medium-angle annular dark-field (MAADF) detectors were 80–300 mrad and 60–80 mrad, respectively. The probe convergence angle was 30 mrad. A Gatan PEELS 666 spectrometer, retrofitted with an Andor iXon 897 electron-multiplying charge-coupled device camera, was used for the EELS experiments^55^. During measurements, the energy dispersion, the beam current and the EELS collection semi-angle were 1 eV per channel, 30 pA, and 35 mrad, respectively. The direct transfer of samples without ambient exposure was enabled using a STEM with a customized sample loading and transfer system^41,42,56^.

### In-situ laser irradiation

Graphene samples were irradiated with a tunable 6 W diode laser (445 nm, Lasertack GmbH) through a viewport in both STEM and UHV sample preparation chambers. The laser used in the experiments was operated at a 10% duty cycle that reduces the laser power to 600 mW to prevent structural damage in graphene and the sample support^41,43^.

### In-situ In deposition

After the first laser irradiation, In was evaporated in-situ using a custom-built preparation chamber (base pressure ~10^−9^ mbar) coupled to the STEM. For the deposition of In onto graphene, a Knudsen cell with In pellets (99.99% purity, Kurt J. Lesker) was heated to 700 °C, while the graphene was kept at room temperature. The nominal thickness of the In measured on quartz microbalance was estimated to be ~10 nm. However, the observed thicknesses of In particles are in the range of 0.9 to 1.6 nm. Having completed the evaporation, the samples were laser irradiated once more to drive In diffusion.

### DFT simulations

Density functional theory (DFT) simulations were performed using the grid-based projector-augmented wave (GPAW) software package^57^. For the relaxation of atomic structures, we used the PBE functional and periodic boundary conditions (with >10 Å of vacuum in the perpendicular direction between the images) in the planewave mode with a cutoff energy of 500 eV and a 5 × 5 × 1 ***k***-point mesh so that maximum forces were <0.02 eV Å^−1,^ ^58^. To study the effect of In decoration on the electronic properties of the GNRs, we calculated their band-structures via spin-polarized DFT. We first converged the ground-state charge density of each system, and to include unoccupied states, we fixed the charge density and doubled the number of bands of which 80% were converged. We calculated the Γ-X band path with 51 ***k***-points, corresponding to the real-space direction along the ribbons. Due to the even number of both In and C atoms in all models, the total magnetic moment was zero and both spin channels were nearly identical.

### STEM image simulations

Independent atom model STEM image (HAADF and MAADF) simulations were performed on the DFT-relaxed models using the QSTEM software with the following experimental parameters: Chromatic aberration coefficient of 1 mm, a spherical aberration coefficient of 1 μm and energy spread of 0.48 eV. The detector semi-angular ranges were set to 80–300 mrad for HAADF and 60–80 mrad for simultaneous MAADF. Similar to the experiment, the probe convergence angle was set to 30 mrad.

### Supplementary information

Supplementary Information

## Data Availability

The authors declare that the data supporting the findings of this study are available within the paper and its supplementary information files.

## References

[1] Wang H (2021). Graphene nanoribbons for quantum electronics. Nat. Rev. Phys..

[2] Wang HS (2021). Towards chirality control of graphene nanoribbons embedded in hexagonal boron nitride. Nat. Mater..

[3] Chamberlain TW (2012). Size, Structure, and Helical Twist of Graphene Nanoribbons Controlled by Confinement in Carbon Nanotubes. ACS Nano.

[4] Zhang T, Wu S, Yang R, Zhang G (2017). Graphene: Nanostructure engineering and applications. Front. Phys..

[5] Geim AK, Novoselov KS (2007). The rise of graphene. Nat. Mater..

[6] Dvorak M, Oswald W, Wu Z (2013). Bandgap opening by patterning graphene. Sci. Rep..

[7] Hu Y (2018). Bandgap engineering of graphene nanoribbons by control over structural distortion. J. Am. Chem. Soc..

[8] Du L, Nguyen TN, Gilman A, Muniz AR, Maroudas D (2017). Tuning the band structure of graphene nanoribbons through defect-interaction-driven edge patterning. Phys. Rev. B.

[9] Chen Y-C (2013). Tuning the band gap of graphene nanoribbons synthesized from molecular precursors. ACS Nano.

[10] Wagner P (2013). Band gap engineering via edge-functionalization of graphene nanoribbons. J. Phys. Chem. C..

[11] Wang S (2016). Giant edge state splitting at atomically precise graphene zigzag edges. Nat. Commun..

[12] Zhang X (2013). Experimentally engineering the edge termination of graphene nanoribbons. ACS Nano.

[13] Son Y-W, Cohen ML, Louie SG (2006). Half-metallic graphene nanoribbons. Nature.

[14] Yamaguchi J (2020). Small bandgap in atomically precise 17-atom-wide armchair-edged graphene nanoribbons. Commun. Mater..

[15] Merino-Díez N (2017). Width-dependent band gap in armchair graphene nanoribbons reveals fermi level pinning on Au(111). ACS Nano.

[16] Chuvilin A (2011). Self-assembly of a sulphur-terminated graphene nanoribbon within a single-walled carbon nanotube. Nat. Mater..

[17] Sarmah A, Hobza P (2018). Sequential BN-doping induced tuning of electronic properties in zigzag-edged graphene nanoribbons: a computational approach. RSC Adv..

[18] Wu M, Pei Y, Zeng XC (2010). Planar tetracoordinate carbon strips in edge decorated graphene nanoribbon. J. Am. Chem. Soc..

[19] Wang Y, Cao C, Cheng H-P (2010). Metal-terminated graphene nanoribbons. Phys. Rev. B.

[20] Zhu Z, Wang D, Zhang ZH, Qiu M (2016). Magnetic structures and magnetic device properties of edge-modified armchair-edged graphene nanoribbons. Carbon.

[21] Wella SA, Hamamoto Y, Suprijadi, Morikawa Y, Hamada I (2019). Platinum single-atom adsorption on graphene: a density functional theory study. Nanoscale Adv..

[22] Dutta S, Pati SK (2008). Half-metallicity in undoped and boron doped graphene nanoribbons in the presence of semilocal exchange-correlation interactions. J. Phys. Chem. B.

[23] Tang Q, Zhou Z, Chen Z (2013). Graphene-related nanomaterials: tuning properties by functionalization. Nanoscale.

[24] Kan E (2010). Ferrimagnetism in zigzag graphene nanoribbons induced by main-group adatoms. Appl. Phys. Lett..

[25] Rigo VA, Martins TB, da Silva AJR, Fazzio A, Miwa RH (2009). Electronic, structural, and transport properties of Ni-doped graphene nanoribbons. Phys. Rev. B.

[26] Yang X (2021). Single-atom catalytic growth of crystals using graphene as a case study. npj 2D Mater. Appl..

[27] Wang H (2012). Interaction between single gold atom and the graphene edge: A study via aberration-corrected transmission electron microscopy. Nanoscale.

[28] Zhao J (2014). Direct in situ observations of single Fe atom catalytic processes and anomalous diffusion at graphene edges. Proc. Natl Acad. Sci..

[29] Hardcastle TP (2013). Mobile metal adatoms on single layer, bilayer, and trilayer graphene: An ab initio DFT study with van der Waals corrections correlated with electron microscopy data. Phys. Rev. B.

[30] Gan Y, Sun L, Banhart F (2008). One- and two-dimensional diffusion of metal atoms in graphene. Micro Nano: No Small Matter.

[31] Kano E, Hashimoto A, Takeguchi M (2017). Opposite effects of Cu and Pt atoms on graphene edges. Appl. Phys. Express.

[32] Wang WL (2014). Direct observation of a long-lived single-atom catalyst chiseling atomic structures in graphene. Nano Lett..

[33] Zan R, Bangert U, Ramasse Q, Novoselov KS (2012). Interaction of metals with suspended graphene observed by transmission electron microscopy. J. Phys. Chem. Lett..

[34] Chen Q (2016). Elongated silicon–carbon bonds at graphene edges. ACS Nano.

[35] Robertson AW (2013). Dynamics of single Fe atoms in graphene vacancies. Nano Lett..

[36] Yeh C-H (2019). Ultrafast monolayer In/Gr-WS2-Gr hybrid photodetectors with high gain. ACS Nano.

[37] Guo W (2021). Atomic indium catalysts for switching CO_2_ electroreduction products from formate to CO. J. Am. Chem. Soc..

[38] Chen X, Zhu H, Zhu J, Zhang H (2023). Indium-based bimetallic clusters anchored onto silicon-doped graphene as efficient multifunctional electrocatalysts for ORR, OER, and HER. Chem. Eng. J..

[39] Westenfelder B (2011). Transformations of carbon adsorbates on graphene substrates under extreme heat. Nano Lett..

[40] Liu Z (2014). In situ observation of step-edge in-plane growth of graphene in a STEM. Nat. Commun..

[41] Elibol K (2021). Single indium atoms and few-atom indium clusters anchored onto graphene via silicon heteroatoms. ACS Nano.

[42] Elibol K (2020). Process pathway controlled evolution of phase and Van-der-Waals Epitaxy in In/In2O3 on graphene heterostructures. Adv. Funct. Mater..

[43] Tripathi M (2017). Cleaning graphene: Comparing heat treatments in air and in vacuum. Phys. Status Solidi (RRL) - Rapid Res. Lett..

[44] Niggas A (2020). The role of contaminations in ion beam spectroscopy with freestanding 2D materials: A study on thermal treatment. J. Chem. Phys..

[45] Wagner P (2013). Stable hydrogenated graphene edge types: Normal and reconstructed Klein edges. Phys. Rev. B.

[46] Leuthner GT, Susi T, Mangler C, Meyer JC, Kotakoski J (2021). Chemistry at graphene edges in the electron microscope. 2D Mater..

[47] Ortolani L (2012). Folded graphene membranes: mapping curvature at the nanoscale. Nano Lett..

[48] Zayed MK, Hegazy MS, Elsayed-Ali HE (2004). Melting and solidification of indium nanocrystals on (002) graphite. Thin Solid Films.

[49] Yamashiro A, Shimoi Y, Harigaya K, Wakabayashi K (2003). Spin- and charge-polarized states in nanographene ribbons with zigzag edges. Phys. Rev. B.

[50] Wakabayashi K, Dutta S (2012). Nanoscale and edge effect on electronic properties of graphene. Solid State Commun..

[51] Schwierz F (2010). Graphene transistors. Nat. Nanotechnol..

[52] Wakabayashi K, Takane Y, Yamamoto M, Sigrist M (2009). Electronic transport properties of graphene nanoribbons. N. J. Phys..

[53] Sevinçli H, Topsakal M, Durgun E, Ciraci S (2008). Electronic and magnetic properties of 3d transition-metal atom adsorbed graphene and graphene nanoribbons. Phys. Rev. B.

[54] Xiao BB, Lang XY, Jiang Q (2014). Pt monatomic wire supported on graphene nanoribbon for oxygen reduction reaction. RSC Adv..

[55] Susi T (2017). Single-atom spectroscopy of phosphorus dopants implanted into graphene. 2D Mater..

[56] Mittelberger A, Kramberger C, Meyer JC (2018). Insights into radiation damage from atomic resolution scanning transmission electron microscopy imaging of mono-layer CuPcCl16 films on graphene. Sci. Rep..

[57] Mortensen JJ, Hansen LB, Jacobsen KW (2005). Real-space grid implementation of the projector augmented wave method. Phys. Rev. B.

[58] Hjorth Larsen A (2017). The atomic simulation environment—a Python library for working with atoms. J. Phys.: Condens. Matter.

[59] Dyck, O., Lupini, A. R. & Jesse, S. Direct-Writing Atom-by-Atom. arXiv (2023) arXiv:2301.02743. 10.1021/acs.nanolett.3c00114 36877825

