# Supplementary material for: Linear indium atom chains at graphene edges
Source: NPJ 2D Mater Appl. 2023 Jan 25;7(1):2. doi: 10.1038/s41699-023-00364-6 (PMC11041670; doi:10.1038/s41699-023-00364-6)
Supplement: Supplementary file 1 — Supplementary Information [file 41699_2023_364_MOESM1_ESM.pdf]

Supplementary Information:

Linear indium atom chains at graphene edges

Kenan Elibol,[1,2,*] Toma Susi,[1] Clemens Mangler,[1] Dominik Eder,[3] Jannik C. Meyer,[1,4] Jani Kotakoski,[1] Richard G. Hobbs,[5,6] Peter A. van Aken,[2] Bernhard C. Bayer[1,3,*]

[1]University of Vienna, Faculty of Physics, Boltzmanngasse 5, A-1090, Vienna, Austria

[2]Max Planck Institute for Solid State Research, Heisenbergstrasse 1, 70569 Stuttgart, Germany

[3]Institute of Materials Chemistry, Technische Universität Wien (TU Wien), Getreidemarkt 9/165, A-1060 Vienna, Austria

[4]Institute for Applied Physics, University of Tübingen, Auf der Morgenstelle 10, 72076 Tübingen, Germany

[5]Centre for Research on Adaptive Nanostructures and Nanodevices (CRANN) and the SFI Advanced Materials and Bio-Engineering Research Centre (AMBER), Dublin 2, Ireland

[6]School of Chemistry, Trinity College Dublin, The University of Dublin, Dublin 2, Ireland

*Corresponding authors: bernhard.bayer-skoff@tuwien.ac.at, k.elibol@fkf.mpg.de

In atoms at the edges of GNRs forming on monolayer graphene

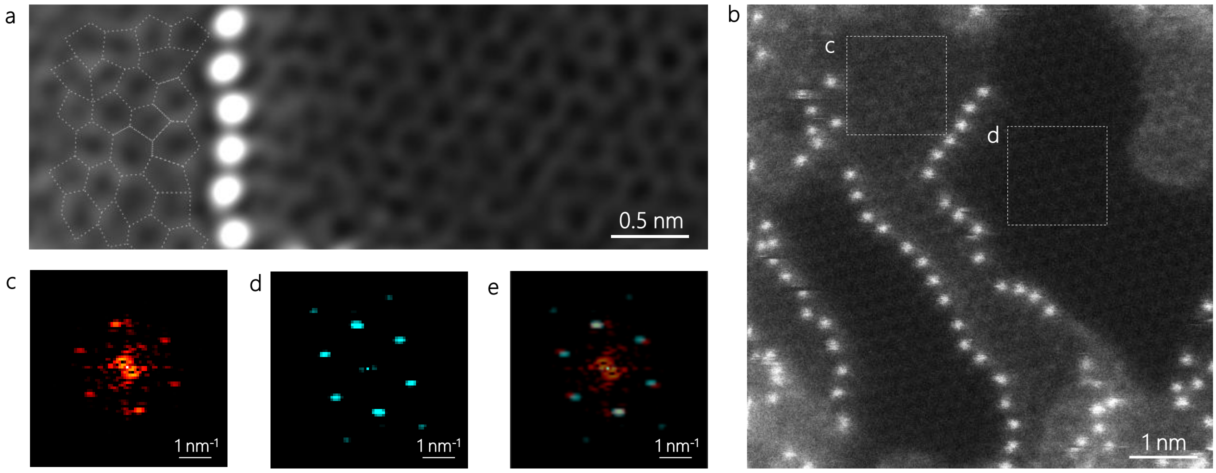

Supplementary Figure 1. In-terminated graphene edge on monolayer graphene. (a) Close-up of MAADF-STEM image of the In-terminated graphene edge on monolayer graphene (between the areas marked c and d in panel (b)). The image is double Gaussian filtered. (b) Overview MAADF-STEM image of an In-terminated GNR. (c,d) FFTs of the areas marked in white dashed frames in panel (a) on GNR and bare graphene support, respectively. (e) Superimposed FFTs shown in panels (c) and (d).

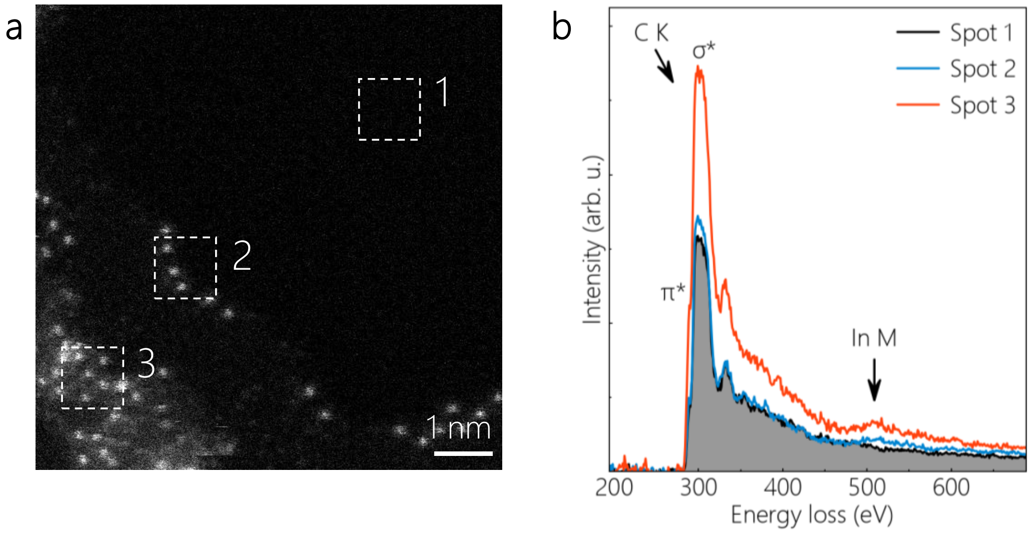

Supplementary Figure 2. EELS characterization for In-decorated graphene edge. (a) HAADF-STEM image of an In-decorated graphene edge. (b) Core-loss EEL spectra summed from different areas of the spectrum image, as shown on the HAADF image in panel (a).

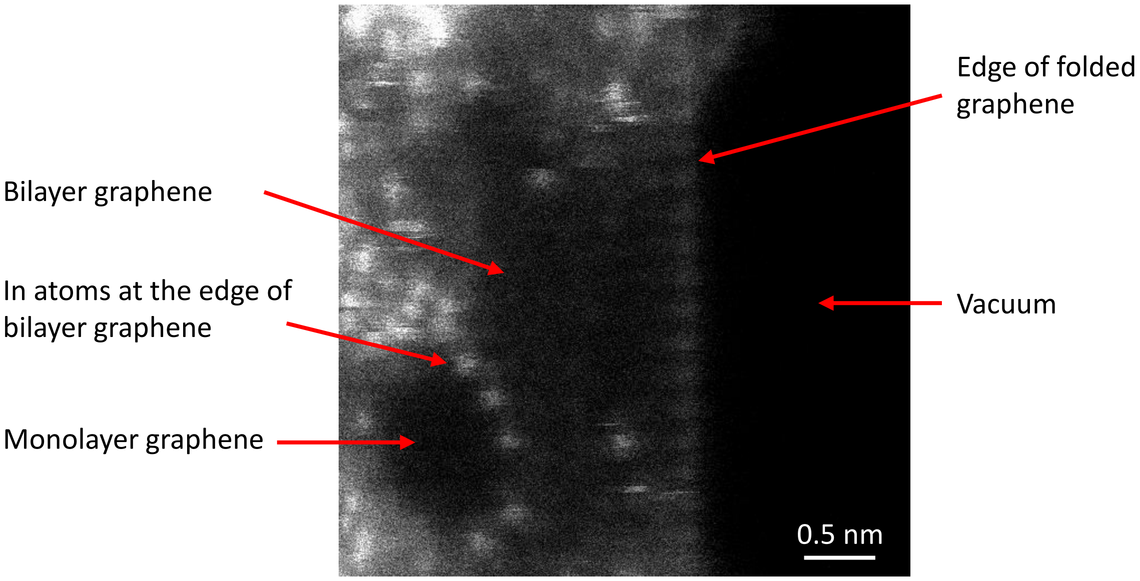

Supplementary Figure 3. Folded graphene. Raw MAADF-STEM image of a folded graphene region.

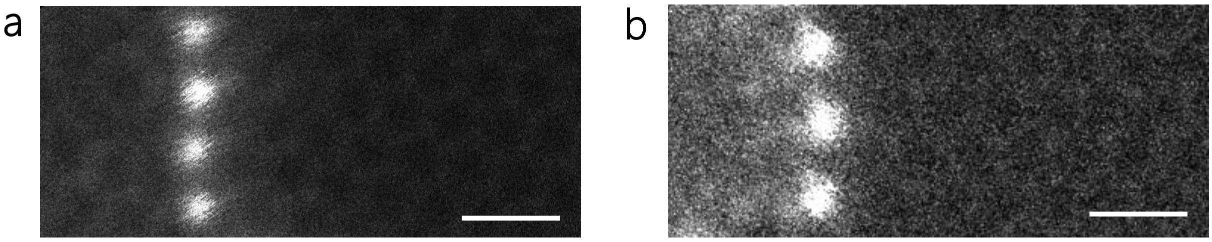

Supplementary Figure 4. In-decorated near-ZZ and near-AC edges. Raw HAADF-STEM images of In-decorated graphene with (a) near-ZZ and (b) near-AC edges.

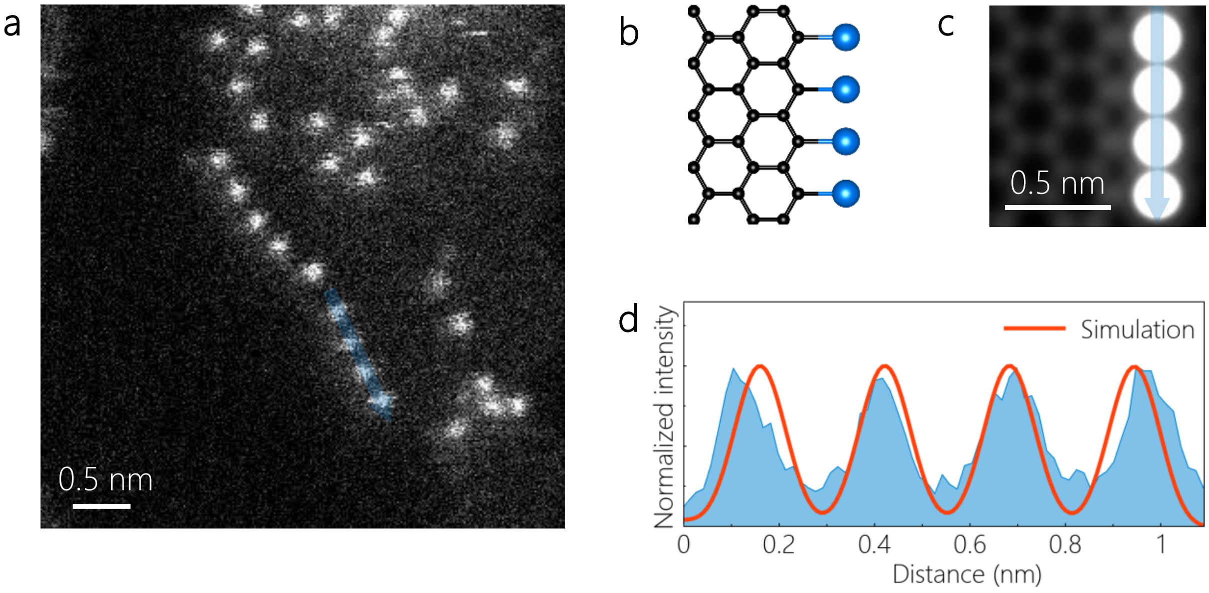

Supplementary Figure 5. In-decorated near-ZZ graphene edge. (a) HAADF-STEM image of an In-decorated near-ZZ graphene edge. (b) DFT-relaxed model of In-decorated ZZ edge of graphene and (c) its corresponding HAADF-STEM image simulation. (d) Intensity profiles measured along the semi-transparent blue lines on panels (a) and (c).

DFT simulations for In-terminated ZZ and AC-GNR edges

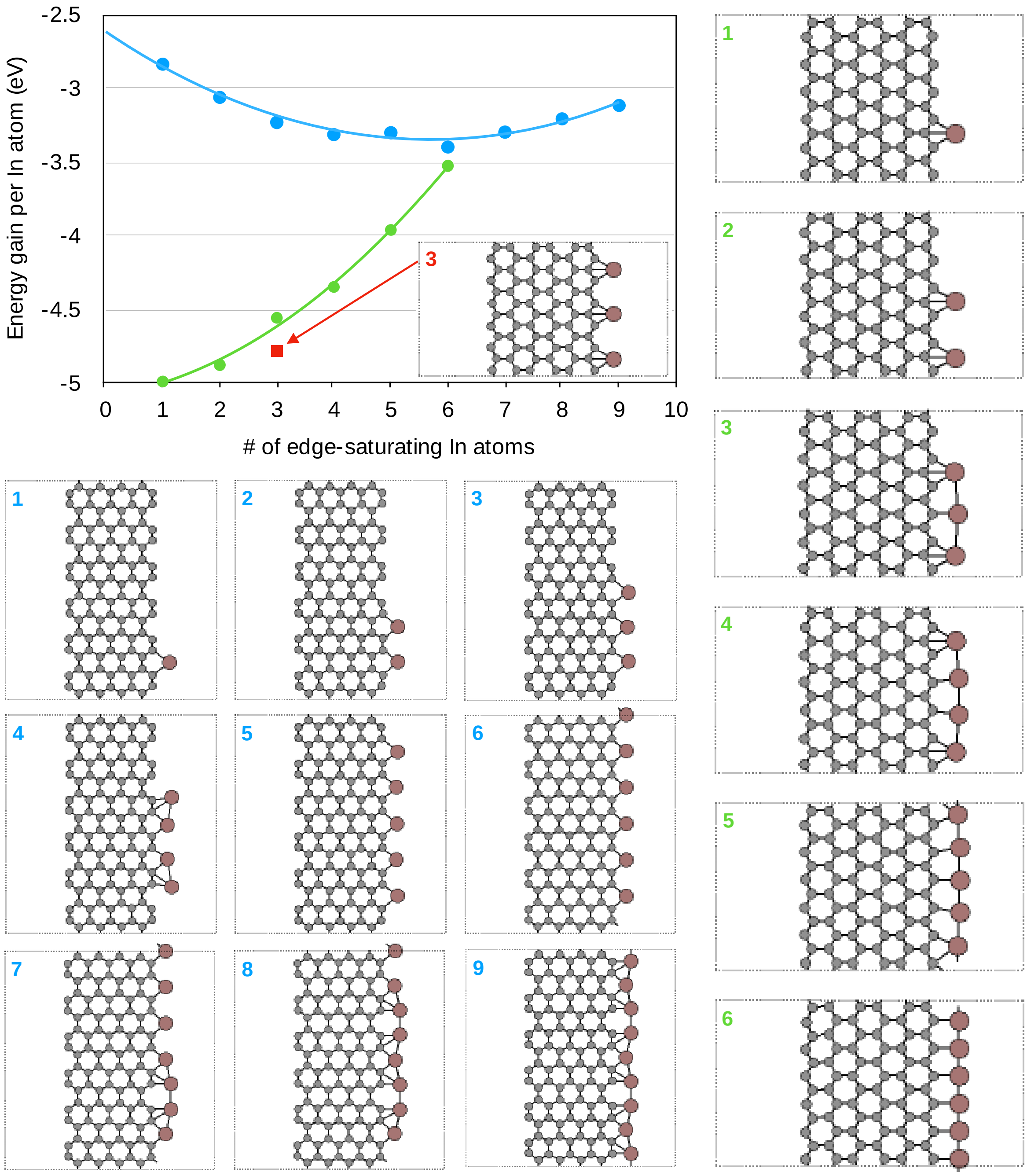

Supplementary Figure 6. DFT simulations for In-decorated ZZ and AC graphene edges. Energy gain per In atom at AC-GNR (N = 9, blue) and at ZZ-GNR (N = 6, green). The inset shows an alternative slightly more stable configuration for three In atoms at the ZZ edge (red). The panels display the DFT-relaxed models (In atoms shown in rose and C in grey). Note that since the initial positions of the sequentially added In atoms were manually chosen, even after careful relaxation these may not necessarily reflect the globally lowest energy configurations.

Supplementary Table 1. DFT-derived energies for In-decorated ZZ edges. Total energy, total energy gain, and energy gain per In atom for the structures including different number of In atoms along the edge of ZZ-GNR (N = 6). Note that since in our DFT code, the energy of an isolated atom in vacuum is zero, these energies correspond to formation energies with a zero chemical potential.

# of In	E_{tot} (eV)	E_{gain} (eV)	E_{gain} (eV) per In
0	-642.457		
1	-629.446	-4.989	-4.989
2	-634.211	-9.753	-4.877
3	-638.131	-13.673	-4.558
4	-641.851	-17.393	-4.348
5	-644.266	-19.809	-3.962
6	-666.538	-42.082	-3.507
3 (alternative) (see Figure S7, red)	-638.743	-14.286	-4.762

Supplementary Table 2. DFT-derived energies for In-decorated AC edges. Total energy, total energy gain, and energy gain per In atom for the structures including different number of In atoms along the edge of AC-GNRs (N = 9).

# of In	E_{tot} (eV)	E_{gain} (eV)	E_{gain} (eV) per In
0	-943.580		
1	-946.419	-2.838	-2.838
2	-949.705	-6.124	-3.062
3	-953.276	-9.695	-3.232
4	-956.838	-13.258	-3.315
5	-960.091	-16.511	-3.302
6	-963.973	-20.393	-3.399

7	-966.667	-23.087	-3.298
8	-969.250	-25.669	-3.209
9	-971.637	-28.056	-3.117

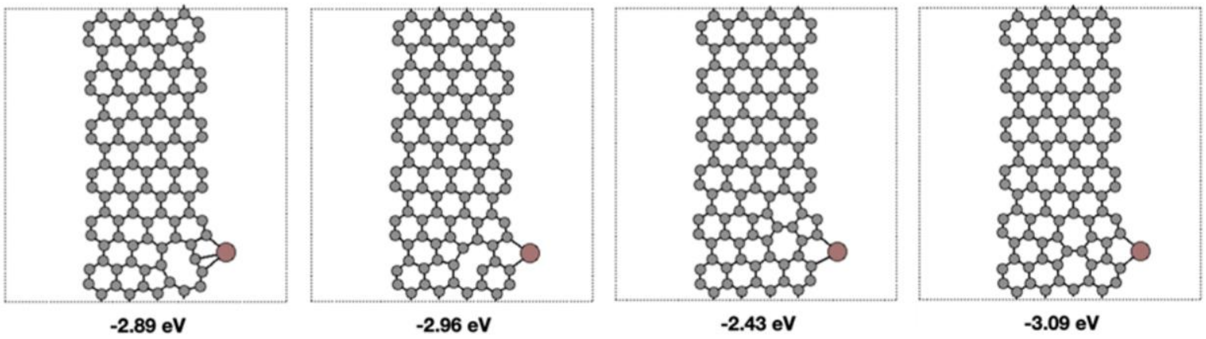

Supplementary Figure 7. DFT simulations for In-terminated AC-GNR edge with defects. DFT relaxed models showing single In atoms attached to the edge of AC-GNR (N = 9) with defects including single removed C atoms (the first two configurations) and Stone-Wales (the last two).
